# Fast Scene Recognition and Camera Relocalisation for Wide Area Augmented Reality Systems

**DOI:** 10.3390/s100606017

**Published:** 2010-06-14

**Authors:** Tao Guan, Liya Duan, Yongjian Chen, Junqing Yu

**Affiliations:** 1 School of Computer Science & Technology, Huazhong University of Science and Technology, No.1037 Luoyu Road, Wuhan 430074, China; E-Mail: qd_gt@126.com; 2 Digital Engineering & Simulation Research Center, Huazhong University of Science and Technology, No.1037 Luoyu Road, Wuhan 430074, China; E-Mails: jessduanjessduan@126.com (L.D.); chyojn@gmail.com (Y.C.)

**Keywords:** augmented reality, wide-area, registration, scene recognition, adaptive random trees

## Abstract

This paper focuses on online scene learning and fast camera relocalisation which are two key problems currently limiting the performance of wide area augmented reality systems. Firstly, we propose to use adaptive random trees to deal with the online scene learning problem. The algorithm can provide more accurate recognition rates than traditional methods, especially with large scale workspaces. Secondly, we use the enhanced PROSAC algorithm to obtain a fast camera relocalisation method. Compared with traditional algorithms, our method can significantly reduce the computation complexity, which facilitates to a large degree the process of online camera relocalisation. Finally, we implement our algorithms in a multithreaded manner by using a parallel-computing scheme. Camera tracking, scene mapping, scene learning and relocalisation are separated into four threads by using multi-CPU hardware architecture. While providing real-time tracking performance, the resulting system also possesses the ability to track multiple maps simultaneously. Some experiments have been conducted to demonstrate the validity of our methods.

## 1. Introduction

The objective of augmented reality (AR) is to add virtual objects to real video sequences, allowing computer-generated imagery to be overlaid on the video in such a manner as to appear part of the viewed 3D scene [1,2]. Applications include computer-aided surgery, robot teleoperation, and special effects for the film and broadcast industries. Registration between real and synthetic worlds is one of the major technological issues in order to create AR systems. As the user moves his/her head or viewpoint, the virtual objects must be properly aligned with the objects in the real world, or the coexistence of the virtual world and the real world will be compromised.

In recent years, registration methods for wide area unprepared environments have attracted much attention [3–6]. These methods have several advantages compared with registration methods which depend on prior knowledge of the user’s environment. For example, tracking is not limited to the prepared scenes, thus, users can walk anywhere they want and superimpose virtual objects dynamically, according to the requirements of the AR applications.

The authors have previously proposed a wide area registration framework based on multiple maps and a natural features tracking technique [6]. This method partitions the whole scene into some geometry independent maps according to the user’s requirements, and all the built maps are integrated into a single tracking system by using a fast image learning and recognition engine. The result is a system that copes with several hundreds of maps at frame-rate, with an agility and scalability rivaling that of single map based systems. While promising, it has some limitations. For example, only two keyframes are used to build the map of each target place. The user’s line of sight is limited to the field covered by these two images. Thus, user’s scope of activities has been greatly restricted.

We can simply use the online structure from motion technique proposed in [4,7] to improve the tracking performance of our previous method. When tracking a map, new keyframes can be added to the system dynamically and newly observed features can be triangulated and optimized subsequently. These reconstructed 3D features can then be added to the tracked map to improve the tracking agility.

While promising, there are some difficulties we must cope with when using online structure from motion technique to improve the performance of our previous method. First, as described in our previous work, an effective scene organizing mechanism is needed to enable the system the ability to learn scenes incrementally and recognize target scenes in real-time. In our previous research, we proposed to solve the above problem by using random trees. However, we have found that the performance of random trees deteriorates markedly with the increase of the scale of each map and the number of local scenes. This is particularly noticeable in our case since several dozens of keyframes are needed for each map to provide flexible tracking performance. In this research, we propose to use adaptive method to generate classification trees dynamically and use Graphics Processing Units (GPU) to accelerate the recognition process. The result is a system whose scalability is significantly better than traditional methods while providing reasonable recognition rates.

Secondly, a fast natural features matching technique together with an effective outliers removing strategy are needed to enable our system with the ability to automatically relocalize from tracking failures. While fast natural features matching and outliers removing are not pivotal problems of our previous research in which each map contains at most hundreds of natural features, this is not the case in our current system since each map may contains thousands of mapped 3D features. In this case, traditional natural features matching and outliers removing strategies may be major obstacles to ensure the real-time performance. In this paper, we use random ferns to generate descriptors of natural features. These descriptors are fast both to compute and to match, while providing the discriminative power that is comparable to that of state-of-the-art methods. We also propose an enhanced PROSAC algorithm to accelerate the process of outliers removing. The algorithm can significantly reduce the computation complexity compared with traditional algorithms, which facilitates the process of online camera relocalisation to a large degree.

Thirdly, in our previous research, real-time performance could be obtained by implementing all the computation steps in a single thread. However, the single thread work mode is not suitable for the research of this paper since both online mapping and scene learning are time-consuming steps. To obtain a system with real-time tracking performance, we implement our algorithms in a multithreaded manner by using a parallel-computing scheme. Camera tracking, scene mapping, scene learning and relocalisation are separated into four threads by using multi-CPU hardware architecture. While providing real-time tracking performance, the result system also possesses the ability to track multiple maps simultaneously.

The rest of this paper is organized as follows: Section 2 is the related work and our contributions. Section 3 presents adaptive random trees based scene learning and recognition methods. Section 4 deals with the problems of natural features matching and camera relocalisation. Section 5 gives the online mapping and camera tracking method. Section 6 deals with our parallel-computing scheme based registration method. Section 7 shows some experimental results. Section 8 discusses some limitations. Section 9 is a conclusion.

## 2. Related Work and Our Contributions

There exists some related research on scene learning and recognition problems for AR applications. Lee *et al.* [8] proposed to solve the online scene recognition problem by matching SIFT features between input frame and previously stored features directly. The scene which has the maximum number of matched features among all scenes is returned as the recognition result if the number of matched features exceeds a certain threshold. While the method is feasible for a system which contains several maps and each has hundreds of features, it is not fast and accurate enough for our research, since our system may contain tens or hundreds of maps each of which holds thousands of mapped 3D features. Klein *et al.* [4,9] proposed to facilitate the scene recognition processes by using keyframes. Each keyframe is represented by a descriptor which will be compared to the input frame’s descriptor by using NCC to find the closest match to assistant the scene recognition processes. Experimental results indicate that the methods can deal with hundreds of keyframes in real-time while spending virtually no time for the learning process. However, this is not enough to meet the requirement of our research since our system may contain thousands or even tens of thousands of keyframes. In our previous research [6], we proposed to deal with scene learning problem by using random trees. Each scene is represented by a predefined numbers of local patches surrounding the SIFT features [10] detected from the two keyframes. These local patches will be used to train the random trees built in advance for online scene recognition use. While promising, we have found that the recognition rates deteriorate obviously with the increase of the scale of each map and the number of local scenes. In this paper, we adjust the classification trees dynamically to improve recognition rates and use GPU to accelerate the recognition process. Compared with our previous work, the method of this paper provides more accurate recognition rates together with the scalability that significantly better than state-of-the-art methods.

Some research has been carried out on the fast camera relocalisation problem. Williams *et al.* [11] designed a fast method to relocalize a monocular visual SLAM system after tracking failure. The monocular SLAM system stores the 3D locations of visual landmarks, together with a local image patch. When the system gets lost, candidate matches are obtained using correlation, and then the pose of the camera is solved via an efficient implementation of RANSAC using a three-point-pose algorithm. Williams *et al.* [12] also proposed to use a randomized lists classifier instead of correlation in their latest research to improve matching performance. Some other similar methods can be found in [13,14]. While feasible, the scalabilities of the above methods are not satisfying since prominent feature point descriptors allow reliable real-time matching but at a computational cost that limits the number of points (less than one hundred) that can be handled on PCs. In this research, we design a scalable camera relocalisation method by using a fast and compacted natural features description method. We also propose an enhanced PROSAC algorithm to accelerate the process of outliers removing. Compared with traditional algorithms, our method can significantly reduce the computation complexity, which enables our system the ability to relocalize the camera from tracking failures rapidly even when the recognized map contains thousands natural features.

There are some researchers who propose to use parallel-computing schemes to realize real-time wide area registration for AR applications. Klein *et al.* [4] proposed the separation of camera tracking and scene reconstruction into two individual tasks, processed in parallel threads on a dual-core computer: one thread deals with the task of robustly camera pose tracking, while the other produces a 3D map of point features from previously observed video frames. This allows the use of computationally expensive bundle adjustment optimization technique not usually associated with real-time operation. In [8] and [15], researchers also propose to speed up natural features (SIFT) detecting and matching processes by using parallel-computing schemes to realize real-time camera tracking for registration use. In this paper, we demonstrate that the tasks of scene learning and camera relocalisation can also be separated as individual threads to further improve tracking performance. Camera tracking, scene mapping, scene learning and relocalisation are split into four threads in our system by using multi-CPU hardware architecture. While providing real-time registration performance, the result system also possesses the ability to track multiple maps simultaneously.

Compared to the authors’ previous work [6], the main contributions of the research reported in this paper can be summarized as follows:

1. We propose to use adaptive random trees to deal with the online scene learning problem. The result is a system whose scalability is significantly better than traditional methods, while providing reasonable recognition rates.
2. We design a scalable camera relocalisation method by using a fast and compacted natural features description method. Moreover, the process of outliers removing is accelerated by an enhanced PROSAC algorithm that can reduce the computation complexity significantly. The resulting system has the ability to recover from tracking failures rapidly, even when the recognized map contains thousands of natural features.
3. We also employ a parallel-computing scheme with multi-CPU hardware architecture to improve tracking performance. We split camera tracking, scene mapping, scene learning and relocalisation into four individual tasks, processed in four parallel threads on a 4-core computer. While providing real-time tracking performance, the result system also possesses the ability to track multiple maps simultaneously.

Subsequent sections describe in detail the method used, present results and evaluate the method’s performance.

## 3. Scene Learning and Recognition Using Adaptive Random Trees

This section gives the adaptive random trees based scene learning algorithm. We first briefly review the method used in our previous work, and then give the detailed description of the ameliorations we made to improve the performance of our previous method.

### 3.1. The Implementation of Our Previous Method

At the center of our previous work is a classifier ℜ constructed by a set of randomized trees. Each internal node of each tree contains a test as Equation (1) that splits the space of data to be classified:

(1) $$Q_{i}=\{\begin{array}{cc} \mathrm{if}\;\;\left(f(a_{i})\geq =f(b_{i})\right) & \mathrm{go to left child}\\ \text{otherwise} & \mathrm{go to right child} \end{array}$$

Each test *Q* simply compares the patch’s gray scale values *f*() at two pixel locations *a* and *b*. The random trees are built in advance by randomly selecting the node tests, *i.e.,* the *a_i_* and *b_i_* in Equation (1).

The leaf nodes of each tree store the number of reached patches of each class as:

(2) $${\hat{N}}_{L}\;(ℜ(f)=c)=n_{c}^{L}$$

where *c* is a class label. $n_{c}^{L}$ is the number of patches of class *c* in the training set that reach the leaf node *L*.

To train the randomized trees when a new map *M*_i_ is added into the system, we firstly get a certain number of local patches {*f_i,1_*, *f_i,2_*, …, *f_i,N_*} from the keyframes used to reconstruct the map. These patches are dropped down each tree according to the binary tests as Equation (1), and then Equation (2) is used to deduce the class *i*’s patch numbers which will be stored in the reached nodes for recognition use.

In the recognition stage, a scene is identified by dropping the detected patches {*f_1_*, *f_2_*, …, *f_N_*} of the input frame down each tree and considering the sum of the patch numbers (subject to a threshold) stored in the leaf nodes they reach as:

(3) $$\mathrm{class}(\{f_{1},\;f_{2},...,\;f_{N}\})=\text{arg}\;\underset{c}{\text{max}}\sum_{i=1}^{N}\;\sum_{t=1}^{T}{\hat{N}}_{L(t,f_{i})}\;(ℜ(f_{i})=c)$$

where *N̂_L_*_(*t*, *f_i_*)_ (ℜ(*f_i_*) = *c*) is the number corresponding to class *c* stored in the leaf *L*(*t, f_i_*) of tree *t* reached by patch *f_i_*, and *t* is a tree label.

### 3.2. Adaptive Random Trees

While feasible, there are some problems we must to deal with when we use the above method to fulfill the scene learning and recognition tasks in this paper. Firstly, we use the sum of the patch numbers as Equation (3) as the recognition method, which is based on the precondition that each scene contains only two keyframes and the patch number is identical for each class. However, the above precondition will not be guaranteed in this research, since different maps may contain different numbers of keyframes which results in the patch numbers for different classes not being identical.

Secondly, the recognition rates of our previous method deteriorate obviously with the increase of the scale of each map and the number of local scenes. This is particularly noticeable in our case since several dozens of keyframes are needed for each map to provide flexible tracking performance.

Thirdly, the scalability is not satisfied since the recognition time grows sharply with the increase of the number of classes.

To solve the first problem, we use posterior probabilities of the original definition of randomized trees [16] as Equation (4) instead of patch numbers to measure the similarity between two local patches:

(4) $${\hat{P}}_{L}\;(ℜ(f)=c)=\frac{n_{c}^{L}/n_{c}}{S_{L}}$$

where *n_c_* is the total patch number of class *c* that is used in the training. $S_{L}=\sum_{c=1}^{C}n_{c}^{L}/n_{c}$ is a normalization term that enforces ∑*_c_* *P̂_L_* (ℜ(*f*) = *c*) = 1.

The recognition method used in this paper can then be given by considering the average of the posterior probabilities as:

(5) $$\mathrm{class}(\{f_{1},\;f_{2},...,\;f_{N}\})=\text{arg}\;\underset{c}{\text{max}}\;\frac{1}{N}\sum_{i=1}^{N}\sum_{t=1}^{T}\;{\hat{P}}_{L(t,\;f_{i})}(ℜ(f_{i})=c)$$

where *P̂_L_*_(*t*, *f_i_*)_ (ℜ(*f_i_*) = *c*) is the posterior probability corresponding to class *c* deduced from the data stored in the leaf *L*(*t, f_i_*) of tree *t* reached by patch *f_i_*.

For the second problem, there are mainly two reasons which cause the deterioration of our previous method’s recognition rates. The first reason is a consequence of the training data. Since we represent local patches by grayscale images, the similarity between different patches will become increasingly evident with the increase in the number of local patches. The second reason is from the method used to build the random trees. Since the random tests of internal nodes are generated in advance and the structures of the built trees can not be changed, the capability to split the space of data can not be adjusted with the change of the training data.

We solve the above two problems as follows:

Firstly, instead of grayscale values, we use HSV values to describe local patches. Compared with grayscale values, HSV gives higher dimensional feature vectors that allow the algorithm to build a classifier able to distinguish between large numbers of classes. Moreover, the HSV representation is more robust to illumination changes than the RGB color space because it tends to largely limit the effects of the most important, practically occurring illumination changes to just one of the three bands.

Secondly, we make use of the adaptive method proposed in [16] to generate node tests and adjust the structures of the random trees dynamically according to the input data. We define two thresholds which will be used in our method. The first threshold is *Num_max_* which denotes the maximum number of local patches that each leaf node holds. The other threshold is *Dep_max_* which denotes the maximum depth of each tree. With the above two thresholds defined, we give the adaptive random trees generating method as follows: when training a local patch, we directly drop it down the random tree by using the random tests as Equation (1) that already exist in the internal nodes. If the total patch number in the reached leaf node exceeds the predefined threshold *Num_max_*, we simply generate a new random test and assign it to the reached leaf node. The local patches in the reached leaf node can then be split into the two new generated leaf nodes (right and left child of the reached leaf node) according to the generated test. The above leaf node splitting process will be repeated for each new generated node until any of the following cases is satisfied:

1. The number of patches in the leaf is smaller than the predefined threshold *Num_max_*.
2. The tree reaches the given depth *Dep_max_*.
3. All patches are constant in the leaf, which means that new partitions can make no changes in the leaf node.

An experiment is carried out on UKBench image database [17] to prove the validity of the above two ameliorations. There are 2,550 classes in UKBench image database, and each class contains four images corresponding to the same scene. The recognition rates are computed as follows:

(Number of correct images in first 4 retrieved images/40,800)×100%

**Figure 1.**
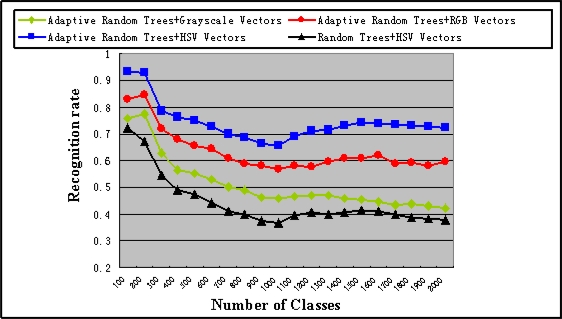
Comparison of recognition rates of different methods.

When using adaptive random trees, we set the two thresholds *Num_max_* and *Dep_max_* to 10 and 30, respectively. When using our previous method, we set the depth of each tree to 15. Ten trees are used in each method and the recognition rates are shown in Figure 1. We can see that the recognition rates are improved obviously with the use of adaptive method and HSV vectors. Moreover, when all the 10,200 images have been trained, the average depth of the adaptive random trees is 13.5 which is still less than the depth of our previous method. These results prove that while not increase the memory consumption, the adaptive method can provide more accurate recognition rate than traditional approach especially with a large number of classes.

**Figure 2.**
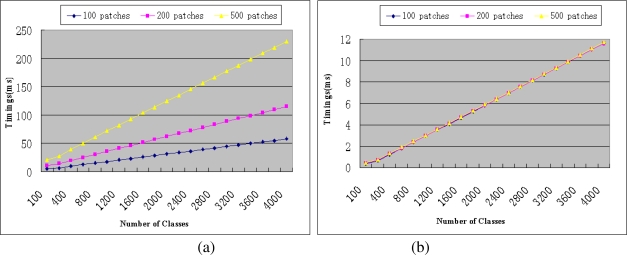
Comparison of the recognition timings between CPU and GPU based methods when using different number of patches.

For the third problem, we use GPU to accelerate the recognition process to get a more scalable system. We do not store the built adaptive random trees in GPU since we cannot ensure that GPU has enough memory to store these trees, whose memory space will change dynamically according to the number of scenes. Instead, we execute node tests on CPU for all the input patches and transfer all the reached leaf nodes to GPU. The posterior probabilities as Equation (4) can then be computed by using CUDA directly. The implementation details of CUDA are out of the scope of this paper, we refer the reader to consult the related literatures [18–20] for advanced details. Figure 2 gives the results to prove the GPU based recognition method’s effectiveness in reducing recognition time. Figure 2a gives the recognition time of a single class with different number of patches when using CPU. Figure 2b is the results when using GPU (NVIDIA GeForce GTX260). We can see that the recognition time of the GPU based method does not increase with patch number and class number markedly and is much less than the time needed in CPU based method. These results convincingly prove that the GPU based recognition method makes the recognition process fast enough for online implementation even with a large number of scenes (classes).

### 3.3. Adaptive Random Trees Based Scene Learning and Recognition

With the above improvements completed, we now present our adaptive random trees based scene learning and recognition algorithms. When a new keyframe belongs to scene (class) *i* is added into the system, we firstly get a set of local patches {*f_i,1_*, *f_i,2_*, …, *f_i,N_*} surrounding the detected natural features. All the HSV vectors converted from these local patches are dropped down each tree according to the binary tests as Equation (1) that already exist in the internal nodes. In this process, if the total patch number in a reached leaf node exceeds the predefined threshold *Num_max_*, we simply use the method discussed in Section 3.2 to split the reached node and adjust the corresponding random tree. With all the patches trained, we use Equation (2) to educe the class *i*’s patch numbers which will be stored in the reached nodes for online recognition use.

In recognition stage, a scene is identified by dropping the HSV vectors of the detected patches {*f_1_*,*f_2_*,…,*f_N_*} in the input frame down each tree. The posterior probability of each class in each reached node is then computed by using Equation (4) and GPU. The recognition result is deduced by considering the average of the posterior probabilities (subject to a threshold) as Equation (5).

## 4. Natural Features Matching and Camera Relocalisation

This section firstly introduces the natural features describing and matching methods used in our research, after which we mainly discuss the enhanced PROSAC method that will be used to realize fast camera relocalisation.

### 4.1. Natural Features Describing and Matching

Recently, a lot of studies [21–24] have been carried out by researchers to design efficient feature describing methods. Among these studies, we have found the descriptors generated by the method of Calonder [21–22] especially suitable for our purpose because these descriptors are fast both to compute and to match, while providing the discriminative power that is comparable to that of state-of-the-art methods.

The method of Calonder [21–22] relies on the fact that if we train a Random Fern (RF) classifier to recognize a number of feature points extracted from an image database, all other points can be characterized in terms of their response to these random ferns. Remarkably, a fairly limited number (500) of base feature points are sufficient.

Descriptors are computed as follows. A set of *B* base feature points are extracted from a representative image and the RF classifier is trained to recognize them under changes in scale, perspective, and lighting. It consists of a set of *N* Random Ferns *F_i_*, where the binary test is a simple comparison of two random locations as Equation (1) in a patch *p* around the feature point. At each leaf of a Fern *F_i_*, there is a vector of responses for all base points, computed from the training set. Let *f_i_(p)* be the vector found by testing the patch *p* through the *F_i_* to a leaf node. The total response vector of *p* is taken to be:

(6) $$r(p)=\prod_{i=1}^{N}\;f_{i}\;(p)$$

The response can be normalized to generate a probability of the patch *p* belonging to any member of the base set. In practice, when *p* belongs to some feature points that are similar to a base keypoint *b*, *r*(*p*) contains high values at *b*’s position in the vector where all others are close to zero. Otherwise, it contains a few relatively large values that correspond to reference feature points that are similar in appearance and small values elsewhere.

For any new feature point *k* not in the base set, the response *r*(*k*) will have high values at locations corresponding to base points that are similar to *k*, and low values elsewhere. Thus, the response vector *r*(*k*) can be considered as the descriptor of feature point *k*.

To compress the descriptors into smaller vectors, Konolige [25] also uses a simple PCA scheme to extract a dense 176-element vector *f_i_*′ that replaces the 500-element *f_i_* on each leaf node. As a further reduction, each element of both *f_i_*′ and the corresponding descriptor *r*′ (*p*) = Π *f*′*_i_* (*p*) are represented by a single byte instead of a floating-point number, thus the descriptors could be compared more quickly by using sum of absolute differences (SAD) [26].

**Figure 3.**
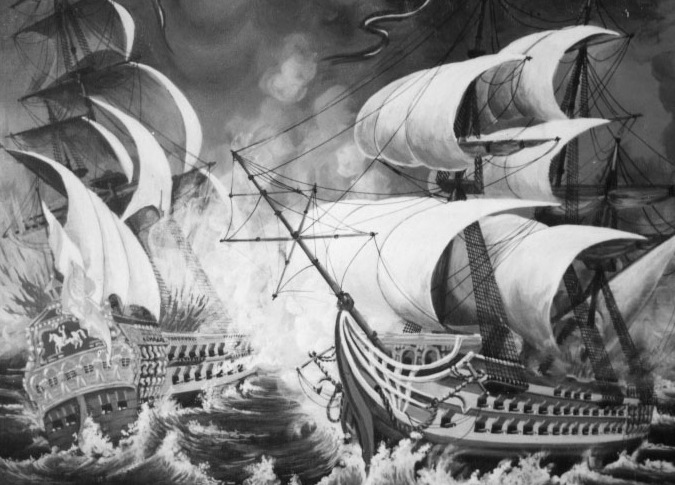
The image we used to train the ferns based descriptor generator.

In our work, we use the image of Figure 3 to obtain examples that will be used to train our descriptor generator. We use a fast corner detector to detect 500 natural features and then implement the above descriptor generating method by using N = 50 binary randomized ferns of depth 10. Experimental results [25] prove that the descriptors generated by this configuration provide a discriminative power that is comparable to U-SURF [23], which is considered as the most efficient robust descriptor. Moreover, we also use GPU and CUDA to accelerate the SAD based matching processes to get a method that is many times faster than traditional approaches. For example, the time for generating 300 descriptors by using dense Random Ferns on a 2.66GHz CPU and matching these descriptors with 5,000 existing descriptors by using GPU (NVIDIA GeForce GTX260) accelerated SAD are 3.7 ms and 6.1 ms respectively. In contrast, the U-SURF will take 70 ms and 350 ms respectively.

### 4.2. PROSAC Based Camera Relocalisation

This section gives the enhanced PROSAC based camera relocalisation method. When a scene has been identified, we use the GPU accelerated SAD and the descriptors generated with the method introduced in the Section 4.1 to get the feature correspondences between the current frame and the recognized scene. The proportion of outliers (mismatches) may be very high in the above obtained matching set since each map may contain thousands of 3D features in our system. In this case, traditional three-point RANSAC may take large numbers of iterations to get a correct camera pose, which will be a main obstacle to fast camera relocalisation.

The standard RANSAC algorithm [27] treats all the matches equally, which means that the random samples are selected uniformly from the whole matching set. It can be considered as a black box that produces *N* tentative matches, *i.e.* the error-prone matches established by comparing local descriptors. In the research of this paper, we find that the matches with higher similarity scores are more likely to be correct matches than the lower ones. Motivated by this property, we use the PROSAC [28] algorithm in which samples are semi-randomly drawn from a subset of the matches with the highest similarity scores, and the size of the hypothesis generation set is gradually increased. In fact, PROSAC is designed to draw the same samples as standard RANSAC algorithm, but only in a different order. The matches more likely to be inliers are tested prior to the others; thereby, the algorithm can arrive at termination criterion and stop sampling earlier.

In PROSAC, The set of *K* potential matches is denoted as *N_k_*. The matches in *N_k_* are sorted in increasing order with respect to the SAD values *s*:

(7) $$n_{i},\;n_{j}\in N_{K}:i<j\Rightarrow s(n_{i})\leq s(n_{j})$$

A set of *k* matches with the lowest SAD values is represented as *N_k_*. Then, the initial subset contains the three top-ranked matches that can be used to compute a candidate camera pose. If all of the samples from the current subset *N_m_* = (*n_1_*,*n_2_*,…,*n_m_*) have been tested and a valid camera pose is not found, then, the next subset is *N_m+1_* = (*n_1_*,*n_2_*,…,*n_m_*,*n_m+1_*), and the following samples consist of *n_m+1_* and the two matches drawn from *N_m_* at random.

**Figure 4.**
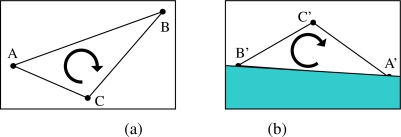
Geometric constraint that must be met by samples when features come from a planar structure. (a) Points A, B and C in a keyframe. (b) Corresponding points A′, B′ and C′ in input frame. Point C′ must not be located in shaded region, since A′, B′, C′ must have the same relative order than A, B, C.

We further improve the performance of PROSAC by checking whether a sample is valid, before actually computing a camera pose. This would avoid estimating the camera pose and searching for inliers, which are by far the two most expensive operations in PROSAC. The checking methods we used are as follows:

1. We reject samples in which two matches come from the same mapped feature or the same detected feature point as these can not produce valid camera poses. We also reject samples in which three matches are collinear or very close to each others in the image as these produce poor pose estimates [12].
2. We reject sets of matches which can not be observed together by a single keyframe. This check prevents attempting to calculate a pose using three features from distant parts of the map which are unlikely all be correct.
3. We also reject samples which do not meet the geometric constraint defined as follows [29]: Given a sample of three matches (*A*′,*A*),(*B*′,*B*),(*C*′,*C*) in which *A,B,C* and *A*′,*B*′,*C*′ are mapped features and detected features respectively. If *A,B,C* come from a planar structure in the real world, it holds that relative order of points *A,B,C* and that of *A*′,*B*′,*C*′ should be the same. Formally, using the vectors *ϕ* = *B* − *A*, *φ* = *C* − *A* and their corresponding vectors *ϕ*′ = *B*′ − *A*′, *φ*′ = *C*′ − *A*′, we express the rule as:
  (8) $$\mathrm{sgn}\left(\left|\begin{array}{cc} {\left(\phi \right)}_{x} & {\left(\phi \right)}_{y}\\ {\left(\varphi \right)}_{x} & {\left(\phi \right)}_{y} \end{array}\right|\right)=\mathrm{sgn}\left(\left|\begin{array}{cc} {\left(\phi^{\prime }\right)}_{x} & {\left(\phi^{\prime }\right)}_{y}\\ {\left(\varphi^{\prime }\right)}_{x} & {\left(\varphi^{\prime }\right)}_{y} \end{array}\right|\right)$$
  where|·| is the determinant function, *(r)_x_* and *(r)_y_* refer to the coordinates *x* and *y* of vector *r* respectively, and sgn(*x*) is the sign function defined as usual. This condition is graphically depicted in Figure 4. We discard all samples which do not hold the above geometric constraint since they will surely lead to an invalid pose under planar structures which exist in man made environments widely.

It is possible that few good samples are thrown away by considering the last two checking methods, but because of the great speed-up they give, many more samples are tested. Besides the above ameliorations, we also set a time limit for the algorithm to insure the continuity of the system. If a valid pose is not obtained by relocalisation thread before the arrival of the next frame. The relocalisation thread will accept the next frame from tracking thread and the recognition algorithm will be run again. We do this to ensure that the obtained inliers can be tracked between consecutive frames in tracking thread for camera pose estimating and registration use.

The completed camera relocalisation method is described as follows:

Step 1: Get feature matches between the recognized scene and the current frame by using SAD and the descriptors generated with the method introduced in the Section 4.1.
Step 2: Sort the feature matches in increasing order with respect to the SAD values.
Step 3: If the remaining time is not enough to carry out a test, go to step 7. Otherwise, generate a sample by using PROSAC.
Step 4: Check whether the generated sample is valid by using the four criterions defined above. If the generated sample is valid, turn to the next step. Otherwise, go to step 3.
Step 5: Compute the candidate pose by using the generated sample and three-point algorithm. Reproject all the 3D features into the current frame by using the computed candidate pose.
Step 6: If the number of inliers is smaller than the predefined threshold, go to step 3. Otherwise, optimize the candidate pose by minimizing the reprojection errors and transfer the optimized camera to the tracking thread for camera tracking use, then turn to the next step.
Step 7: Wait for the next recognized scene and repeat the above steps.

## 5. Online Mapping and Camera Tracking

While online mapping and camera tracking are not the focuses of this paper, we still briefly introduce the methods we used for the sake of integrality.

### 5.1. Online Mapping

We use the method proposed in our previous work [6] to perform system initialization. The method requires users to choose four pairs of corresponding points manually in the two reference images respectively, and then system can calibrate these points automatically to define the position of the virtual object. When tracking a map, new keyframes are added to the system conditionally to allow the map to grow. Once a new keyframe is added, feature correspondences between this keyframe and its closest keyframe are established by using epipolar search. With the matches set obtained, we calculate the 3D positions of the newly observed features by using triangulation, and carry out an optimization process by using local bundle adjustment [4,7] in which only a subset of cameras and 3D points are optimized to improve mapping accuracy. The optimized 3D features can then be added to the system for camera tracking use. When local bundle adjustment has been finished and no new keyframes are inserted, the global bundle adjustment considering all cameras and points is carried out to further improve accuracy. This process can be interrupted when new keyframes is added to the map, so that newly observed features can be integrated into the tracking system within shortest possible time. We also stipulate that new keyframe can be added only when the time since the last keyframe was added exceeds the predefined interval (200 ms in our research). This is to ensure that the system has enough time to finish previous local bundle adjustment.

### 5.2. Camera Tracking

Once a successful relocalisation has been done, the next step is to track the natural features in the input video sequence to compute camera poses for registration use. To find a single mapped feature in the current frame, a fixed-range patch search surrounding the feature’s predicted image location is carried out. To perform this search, the corresponding patch is first warped by using affine transform to take account of viewpoint changes between the patch’s first observation and the current camera position. With the feature matches obtained, we then use the Tukey biweight estimator [30] and Levenberg-Marquardt algorithm to eliminate outliers and compute the camera pose simultaneously.

## 6. Registration Based On Multithreaded Approach

**Figure 5.**
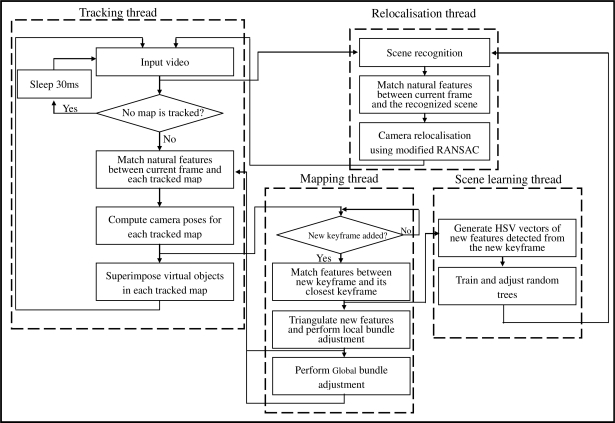
Multithread based registration method.

This section introduces our multithread based registration method of which the flowchart is shown in Figure 5. As can be seen from the figure, camera tracking, scene mapping, scene learning and relocalisation are separated into four parallel threads. Tracking thread copes with the tasks of camera poses estimation and virtual objects augmentation. Mapping thread receives keyframes to build maps. Scene learning thread is used to train and adjust the random trees. Relocalisation thread deals with the tasks of scene recognition and camera pose relocalisation.

We build a randomized ferns based descriptor generator in advance by using the method discussed in Section 4.1. This generator will be used in all the experiments described in this paper. When the system is started, an initialization stage is carried out to generate the random trees to be used for online scene learning and recognizing. We do this by generating ten trees each of which contains only one random test and two leaf nodes. The two thresholds *Num_max_* and *Dep_max_* are set to 10 and 30 respectively to enable the trees to expand dynamically in online stage.

The tracking thread mainly deals with the problems of camera tracking and virtual objects augmentation. It receives input video from the camera and relocalisation results from the relocalisation thread. For each input frame, it matches natural features between current frame and all the tracked maps. Then, camera pose for each tracked map can be computed individually for augmentation purpose by using the method discussed in Section 5.2. In the case where no map is tracked, a delay of 30 ms is taken before the input of the next frame. We do this to allow the relocalisation thread has adequate time to relocalize the first map. We also stipulate that at most three maps can be tracked simultaneously to ensure the real time performance of our system.

Mapping thread accepts keyframes from the tracking thread and performs optimization processes to reconstruct new features. Once a new keyframe is added, we first triangulate the newly observed features and then perform a local bundle adjustment in which only a subset of cameras and 3D points are considered. With the local bundle adjustment converged, the tracked map will be expanded directly by adding the new 3D features to improve tracking agility. When local bundle adjustment has been finished and no new keyframes are inserted, we then perform a global bundle adjustment to further improve accuracy. However, the global bundle adjustment can be interrupted by the arrival of new keyframes. This is to ensure new 3D features can be used in tracking thread within shortest possible time. In case of tracking multiple maps, the mapping thread will continue to expand the map that currently being processed until the tracking in this map is cancelled (manually or automatically) or the map reaches the predetermined number of keyframes. With the above process completed, the tracking thread will turn to expand another tracked map which has the least number of keyframes. The reason why we do this is to give the weakest map the priority to expand to improve the overall performance of our system.

Scene learning thread deals with the tasks of training and adjusting the random trees by using input keyframes. When a new keyframe is added into the current map, we firstly get the set of local patches surrounding the new observed features. These local patches will be converted into HSV vectors for training use. We drop each HSV vector down each tree according to the binary tests that exist in the internal nodes. If the total patch number in a reached leaf node exceeds the predefined threshold *Num_max_*, we simply use the method discussed in Section 3.2 to split the reached node. With all the HSV vectors trained, we make use of Equation (2) to compute the patch numbers which will be stored in the reached nodes for online recognition use.

The relocalisation thread receives input frames from the tracking thread and copes with the problems of scene recognition and camera relocalisation. For each input frame, the HSV vectors of the detected patches are simply dropped down each tree and the posterior probability of each class in each reached node is computed by using Equation (4) and GPU. The scene that has the greatest average probabilities (returned by Equation 5) and is not currently being tracked by tracking thread will be returned for camera relocalisation use. With a scene recognized, the next step is to compute the initial camera pose for tracking use. We firstly generate feature descriptors of the current frame by using the built ferns as discussed in Section 4.1. Then, the matches set between the current frame and the recognized scene will be established by using GPU accelerated SAD. With the matches set obtained, the enhanced PROSAC algorithm discussed in Section 4.2 will be used to compute the initial pose that will be passed to tracking thread for camera tracking and augmentation use. All the above computations should be accomplished before the arrival of the next frame. A time limit computed from the frame rate of tracking thread is set for the enhanced PROSAC algorithm. If the correct pose is not found within this time limit then the algorithm gives up: a new frame is taken from the tracking thread and the algorithm is run again. We do this to ensure that the camera pose will not be too far out of date when found.

In our system, the tracking thread runs as the main thread, while the other three threads run as background processes and provide services to the main thread. Since these threads share some common data such as 3D maps and random trees, we must to deal with these data carefully in each thread to ensure the accuracies of various calculations. For example, the random trees will be visited by relocalisation and learning threads at the same time frequently. The recognition results will be unreliable if we allow the learning thread to adjust the random trees when these trees are being used by relocalisation thread for scene recognition purpose. We solve this problem by storing the adjusted leaf nodes in the learning thread’s local variables which will be used to update the random trees when the relocalisation thread performs feature matching and camera pose computation operations. Similarly, the new 3D features will be stored in the mapping thread’s local variables, and the map will be updated when it is not used by the tracking and relocalisation threads.

## 7. Experiments

The experiments are performed on a computer with a 4-core Xeon 2.66 GHz CPU and 4G RAM, using a Logitech Quick-Cam Pro 9000 video camera with 640 × 480 resolution. Intrinsic camera parameters are calibrated in a one-time offline step using a 6 × 8 checkerboard pattern and the OpenCV implementation in [31].

### 7.1. Mapping and Tracking Results

We build a system using our algorithm to prove the usability of the proposed method for wide area AR applications. The built system contains seven indoor maps and five outdoor maps. The seven indoor maps are built around our laboratory, the area of which is about 80 square meters. The five outdoor maps are built around our campus. Each map contains 21 to 92 keyframes and 1,392 to 4,215 map points, which added up to a total of 725 keyframes and 25,251 3D points.

**Figure 6.**
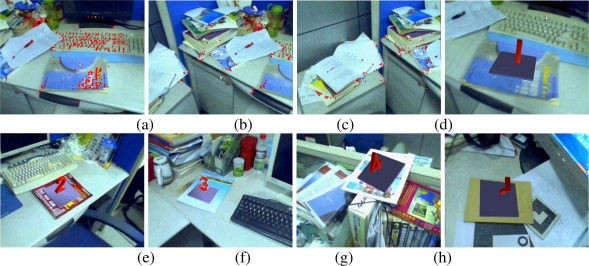

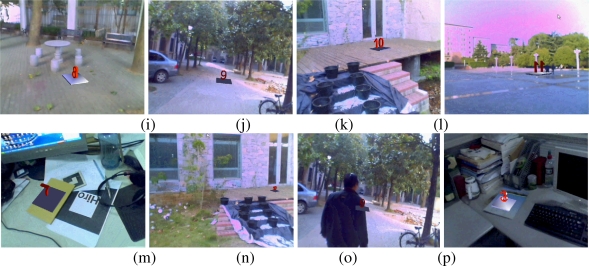
Mapping and augmentation results.

Figures 6a,b,c show the results of camera tracking and map building processes of the first indoor map. Figures 6c–Figure 6l show the augmentation results when tracking some of the built maps. The virtual objects are the 3D words indicating the map number. Figure 6m and Figure 6n are augmentation results of view angles and volume changes when tracking the 7th and 10th maps respectively. Figure 6o gives the augmentation results in case of occlusion while tracking the 9th map. Figure 6p is the augmentation result of illumination changes when tracking the 3rd map. The above results convincingly demonstrate the validity of the proposed online scene reconstructing and camera tracking method.

**Figure 7.**
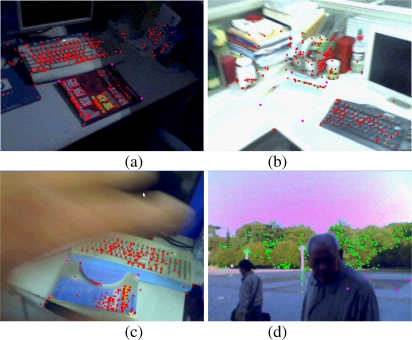
Relocalisation results with occlusions and illumination changes.

The robustness of the proposed scene recognition and camera relocalisation methods to occlusion and illumination changes is illustrated in Figure 7. Figure 7a and Figure 7b give the relocalisation results of the 2nd and 3rd maps respectively with illumination changes. Figure 7c and Figure 7d give the relocalisation results of the 1st and 12th maps respectively in case of occlusions. The above results soundly prove the robustness of the proposed scene recognition and camera relocalisation methods to occlusion and illumination changes.

**Figure 8.**
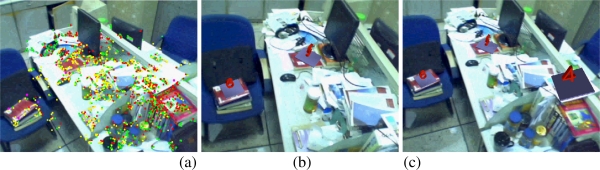

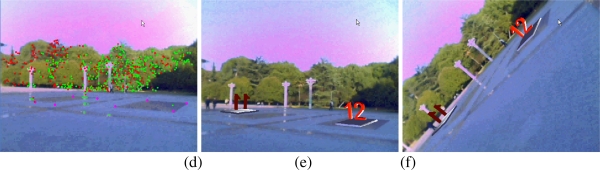
Multiple maps tracking results.

The capability of tracking multiple maps simultaneously is illustrated in Figure 8. Figure 8a gives the tracked features of the 4th (red points), 5th (yellow points) and 6th (green points) maps. Figure 8b and Figure 8c give the augmentation results when tracking two and three indoor maps respectively. Figure 8d gives the tracked features of the 11th (red points) and 12th (green points) maps. Figure 8e and Figure 8f give the augmentation results when tracking these two outdoor maps. The above results effectively prove the validity of the proposed method to deal with multiple maps tracking problem.

### 7.2. Computation Time for Each Thread

Each thread’s computation time of the experiment discussed in Section 7.1 is also recorded. Table 1a gives the computation time of tracking thread when only one map is tracked. The total time is about 60 ms, in which a delay of 10 ms is taken to give relocalisation thread enough time to relocalize another map. Thus the frame rate is 16.7 fps when tracking a single map. The time for tracking two maps is about 65 ms and the corresponding frame rate is 15.3 fps. The time for tracking three maps is about 81 ms and the corresponding frame rate is 12.3 fps.

Table 1b gives the computation time of relocalisation thread when no or only one map is tracked. The time for scene recognition and feature matching is about 14 ms. Iterative pose optimization takes about 5 ms. We give 30 ms to the enhanced PROSAC algorithm to perform outliers removing processes. Thus, it takes about 49 ms to relocalize a new camera when no or only one map is tracked (as discussed in Section 7.5, we left 11 ms to scene recognition process to deal with scene number increase). Since the time for tracking two maps is about 65 ms, we can then alot more time (35 ms) to the enhanced PROSAC algorithm, thus it takes about 54 ms to relocalize the third camera in case of tracking two maps.

Table 1c gives the computation time of the mapping thread. The maximum time to map the new features by using local bundle adjustment is about 160 ms. Since we stipulate that new keyframe can be added only when the time since the last keyframe was added exceeds the predefined interval 200 ms, thus new 3D features can always be added to our tracking system before the input of the next keyframe. The time for global bundle adjustment varies with map size obviously. It takes about 1.7 s for our largest map which contains 92 keyframes and 4,215 3D features to converge. A practical limit to insure good usability for our system is about 100 keyframes for each single map.

Table 1d gives the computation time of the scene learning thread. The maximum time recorded for learning a keyframe is about 122 ms, which is fast enough to allow a reasonable rate of map exploration.

### 7.3. Performance of the Enhanced PROSAC Algorithm

**Figure 9.**
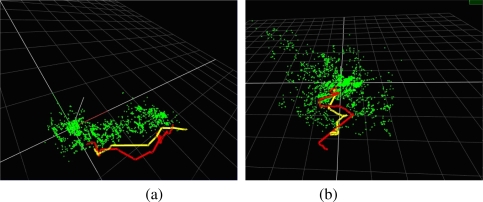
Maps and trajectories used to test the performance of the enhanced PROSAC algorithm.

In this section, we carry out two experiments to compare the enhanced PROSAC algorithm with other methods [27–28,32–36]. To perform the first experiment, we first build a map containing 35 keyframes and 2,102 3D features. As shown in Figure 9a, the green points are the mapped 3D features and the yellow line segment is the camera trajectory. We then capture a video sequence containing 500 frames by moving the camera along the trajectory shown by the red line segment. This video sequence will be used to test the performances of different outliers removing methods. For each frame we give 30 ms to each method to perform outliers removing operation. The numbers of successful relocalisation times in these 500 frames of different methods will be compared. The map used in the second experiment is shown in Figure 9b. It contains 41 keyframes and 3,751 3D features. The second test video also contains 500 frames.

The recorded numbers of successful relocalisation times of different methods in these two experiments are given in Table 2. We can see that the number of successful relocalisation times of our method is far more than other methods’. These results prove that the enhanced PROSAC algorithm can get significant improvements in speed over existing methods, thus is more suitable for use in real-time applications with a limited time budget.

### 7.4. Performance of the Ferns Based Features Describing Method

**Figure 10.**
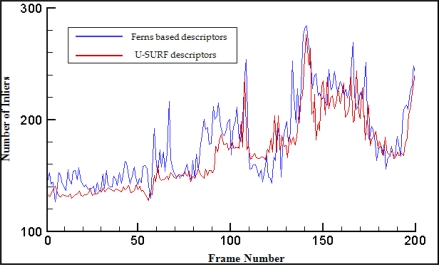
Comparing of matching performance between U-SURF and ferns descriptors.

We also carry out an experiment to compare the match rates of U-SURF descriptors and ferns based descriptors by using the vidoe sequence from the first scene of the experiment given in Section 7.1. We generate the ferns based descriptor by using N = 50 binary randomized ferns of depth 10 and then use these descriptors to establish matches between the modeled scene and the input sequence images. In parallel, we compute U-SURF descriptors for the keypoints extracted from the modeled scene and match each of them against the keypoints in the sequence images by selecting the one which has the nearest U-SURF descriptor. We retain the 1,437 strongest keypoints in the modeled scene and 400 keypoints in the sequence images for the two methods. Then in both cases we use our modified PROSAC estimation to compute the camera pose, which is then refined using a non-linear estimation method using all matches that are compatible with it. All matches are checked against this pose and those whose reprojection using this pose is within 10 pixels are retained as inliers. The graph of Figure 10 depicts the number of correct matches obtained by both methods for all frames in the sequence. Despite their simplicity, ferns based descriptors match at least as many points as U-SURF descriptors and often even more.

### 7.5. Comparison with Other Methods

This section compares the results obtained using the proposed method with other methods. The comparisons made are (1) comparison with the authors’ previous method [6] and (2) comparison with the PTAM (Parallel Tracking and Mapping) [4].

**Figure 11.**
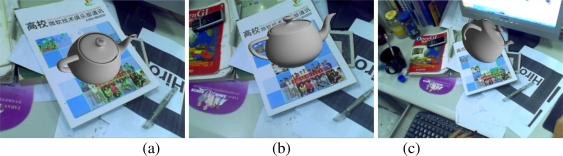

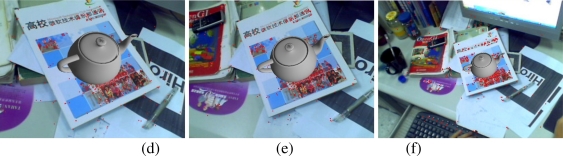
Results of the first experiment used to compare the multiple keyframes based method with two keyframes based method.

**Figure 12.**
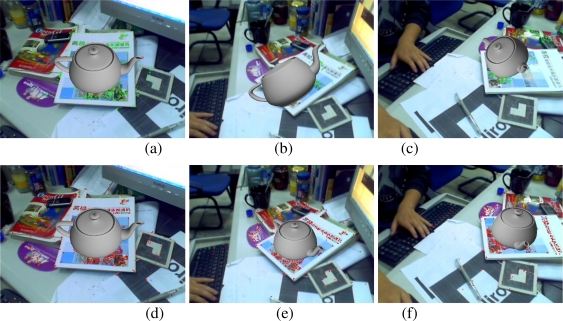
Results of the second experiment used to compare the multiple keyframes based method with two keyframes based method.

**Figure 13.**
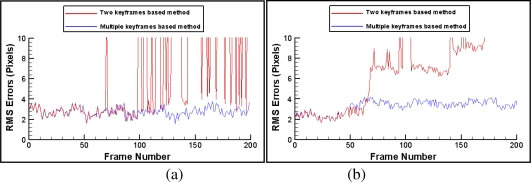
Comparison of RMS errors.

Firstly, we compare our multiple keyframes based tracking method with the method based on two keyframes reported previously by the authors [6]. Figures 11 and 12 give the registration results of the above two methods when moving camera along two different trajectories. Figures 11a,b,c and Figures 12a,b,c show the results using the two keyframes based method. Figures 11d,e,f and Figures 12d,e,f are the results using our multiple keyframes based method. The recorded RMS errors of these two experiments are shown in Figure 13. We can see that tracking accuracy is improved obviously by adding new keyframes and mapped features to tracking system dynamically. These results convincingly prove the effectiveness of using online mapping to improve tracking accuracy.

We also carried out an experiment to compare our method with the PTAM [4] by using the video sequence recorded from the experiment discussed in Section 7.1. We test this sequence using the publicly accessible code PTAM (http://www.robots.ox.ac.uk/~gk/PTAM/). The input frame rate is set to 8 fps to give enough time to the local bundle adjustment thread, and the sequence is repeated for the global bundle adjustment to converge. Even so, we find PTAM can only succeeded in mapping and tracking about half part of the indoor scenes, and failed when the keyframes number exceeds 195. In contrast, as discussed in Section 7.1, our multiple maps based method can provide a satisfying tracking and mapping experience. These results prove that our method is more flexible than PTAM especially with large scale scenes because to build a single map for such a workspace requires computationally expensive global bundle adjustment which cannot be done online.

### 7.6. Scalability

There are mainly two factors that limit the scalability of our method. The first factor is the maximum keyframe number that each map can accommodate, while allowing a reasonable rate of exploration. The second factor is the maximum scene number the method can deal with while maintaining the speed of the camera relocalisation.

As discussed in Section 7.2, since the global bundle adjustment is very time-consuming with large numbers of keyframes, we stipulate that each map contains at most 100 keyframes to ensure that the global bundle adjustment can be completed within a limited time. This enables the tracking thread to obtain 3D features timely and accurately to insure good tracking performance.

The maximum scene number that our system can deal with depends on the time left to the adaptive random trees based scene recognition process. As discussed in Section 7.2, in order to accomplish the camera relocalisation before the arrival of the next frame, we stipulate that the feature detecting and scene recognition time should not exceed 14 ms. Removing the time for feature detecting and HSV vectors preparation (3 ms), the times left to scene recognition is about 11 ms. According to the computation time of GPU accelerated scene recognition algorithm given in Figure 2, our system can manage about 3,800 maps for wide area AR use and the scalability is significantly better than our previous method which can only deals with about 650 maps.

## 8. Discussion

In this paper, we implement a flexible multithread based registration method for wide area AR systems by using online scene mapping and learning techniques. The proposed method can work in any environment without the need to prepare the scene in advance. Moreover, multiple maps can be tracked simultaneously in real time which really enhances the usability of the proposed method.

Real-time implementation is an important issue for wide area AR systems. The proposed method can meet real-time performance requirements because time-consuming steps such as mapping and learning are separated as background processes to reduce the computation complexity of tracking thread. Moreover, we also separate camera relocalisation task from tracking thread to further improve tracking performance. While leaving more time for tracking thread to deal with multiple maps tracking problem, our method also gives relocalisation thread enough time to perform outliers removing processes to recover the lost camera even when the recognized map contains thousands natural features.

In case of tracking multiple maps, our current method can only expand a single map at the same time. Tracking failures in other tracked maps which are currently not being expanded cannot be avoided especially when camera moves out of the scopes covered by these maps. The above problem weakens the usability of the proposed registration method to some degree. Merging all the tracked maps into a single larger map may be an effective method to solve this problem. Developing an online merging algorithm that can integrate all the tracked maps into a single map to further improve the tracking performance of current system is left for our future work.

Modern mobile phones have become a compelling platform for mobile Augmented Reality [36–37]. They contain all the equipment typically required for video based AR. A lot of researches have been carried out to implement natural features matching [38], online mapping [39] and hardware based localization [40,41] to realize real time pose estimation on mobile devices. By contrast, little attention has been paid to realize online scene learning and recognition on mobile phones for wide area mobile AR use. We think that adaptive random trees will provide a good solution to solve the above problem. However, the main obstacle is that they use very large amounts of memory (For example, the classifier in the system described in Section 7.1 will take about 96 M), which makes them impractical for implementation on low-memory mobile devices (These devices do usually not allow more than about 5–10 MB per application). In our future work, we will design a flexible compressing algorithm to reduce the memory usage, by which to facilitate the implementation of adaptive random trees on low-memory mobile devices.

## 9. Conclusions

This paper deals with on online scene learning and fast camera relocalisation problems that currently limit the usability of multi-maps based wide area registration systems. Firstly, we use adaptive method to adjust random trees dynamically and use GPU to accelerate the recognition process. The result is a system whose scalability is significantly better than traditional methods, while still providing reasonable recognition rates. Secondly, we design a scalable camera relocalisation method by using the enhanced PROSAC algorithm that can reduce the computation complexity significantly. The result system has the ability to recover from tracking failures rapidly even when the recognized map contains thousands of natural features. Thirdly, we implement our algorithms in a multithreaded manner by using a parallel-computing scheme. While providing real-time tracking performance, the result system also possesses the ability to track multiple maps simultaneously. In future work, we will design a flexible compressing algorithm to facilitate the implementation of adaptive random trees on low-memory mobile devices.

## Figures and Tables

**Table 1.** Computational timings.

(a)	

**Tracking thread**	

Video capture	33 ms
Feature projection and match	12 ms
Iterative pose optimization	5 ms
Sleep	10 ms

**Total**	60 ms

(b)	

**Relocalisation thread**	

Feature detecting and scene recognition	4 ms
Descriptors generating and matching	10 ms
Modified RANSAC	30 ms
Iterative pose optimization	5 ms

**Total**	49 ms

(c)	

**Mapping thread**	

Feature detecting and epipolar searching	10 ms
Local bundle adjustment	150 ms

**Total**	160 ms

(d)	

**Scene learning thread**	

Keyframe preparation	1 ms
Learning	121 ms

**Total**	122 ms

**Table 2.** Comparison of successful relocalisation times.

	**RANSAC[27]**	**Td,d Test[32]**	**Preemptive[33]**	**Bail-out[34]**	**Wald [35]**	**PROSAC[28]**	**Enhanced PROSAC**
**Video 1**	54	112	153	185	225	302	**417**
**Video 2**	76	151	167	201	247	363	**448**
